# Development of an expert-annotated chest X-ray dataset to support AI validation in tuberculosis diagnosis

**DOI:** 10.1186/s13244-026-02334-0

**Published:** 2026-07-10

**Authors:** Wiwatana Tanomkiat, Shiva Raj Timsina, Thammasin Ingviya, Sitthichok Chaichulee, Patinya Yutchawit, Chiawchan Thuncharoenkanka, Wuttikrai Pannuan, Chureerat Chantharat, Urasri Kerdmeesup, Nantaka Kiranantawat, Sitang Nirattisaikul, Sutarat Tungsagunwattana, Krisna Dissaneevate, Warawut Sukkasem, Chanavee Toh, Suthinee Teerajaruwat

**Affiliations:** 1 Division of Diagnostic Imaging, Department of Radiology, Faculty of Medicine, Prince of Songkla University, Hat Yai, Songkhla, Thailand; 2 Department of Radio-Diagnosis and Imaging, Samtse General Hospital, Samtse, 22001 Bhutan; 3 Department of Family and Preventive Medicine, Department of Clinical Research and Medical Data Science, Faculty of Medicine, Prince of Songkla University, Hat Yai, Songkhla, Thailand; 4 Department of Biomedical Sciences and Biomedical Engineering, Faculty of Medicine, Prince of Songkla University, Hat Yai, Songkhla, Thailand; 5 Department of Internal Medicine, Faculty of Medicine, Mahasarakham University, Maha Sarakham, Thailand; 6 Radiology Department, Udonthani Hospital, Udon Thani, Thailand; 7 Radiology Department, Chiangrai Prachanukroh Hospital, Chiang Rai, Thailand; 8 Tuberculosis Division, Department of Disease Control, Ministry of Public Health, Bangkok, Thailand; 9 Central Chest Institute of Thailand, Department of Medical Services, Ministry of Public Health, Nonthaburi, Thailand; 10 Rajavithi Hospital, Department of Medical Services, Ministry of Public Health, Department of Radiology, College of Medicine, Rangsit University, Bangkok, Thailand; 11 Department of Diagnostic and Therapeutic Radiology, Faculty of Medicine Ramathibodhi Hospital, Mahidol University, Bangkok, Thailand

**Keywords:** Tuberculosis, Chest X-ray, B readers, Inter-rater agreement, Artificial intelligence

## Abstract

**Objectives:**

To assess inter-rater agreement and diagnostic performance of six United States National Institute for Occupational Safety and Health-certified B readers (physicians certified in standardized chest X-ray (CXR) classification for pneumoconiosis) in diagnosing tuberculosis (TB) on CXR, with the goal of developing a reliable dataset to support external validation of artificial intelligence (AI) models for TB detection.

**Materials and methods:**

1097 CXRs from five institutions were analyzed by six B readers. Patients aged ≥ 15 years were included, excluding those with HIV or opportunistic infections. CXRs were classified as unremarkable or abnormal. Abnormalities were categorized using a modified International Labor Organization classification and classified as consistent or inconsistent with TB. Microbiological references included sputum smears, cultures, or molecular tests. Descriptive statistics summarized the CXR and microbiological findings. Inter-rater agreement was assessed with Fleiss’ kappa. Diagnostic performance was evaluated by comparing CXR findings to microbiological references.

**Results:**

Of the 3117 readings, 69% of CXRs were abnormal, and 31% were unremarkable. Microbiological results confirmed that 87% of abnormal CXRs were TB cases, while 83% of unremarkable CXRs were non-TB. Inter-rater agreements were κ = 0.83 for all findings, κ = 0.67 for findings consistent with TB, and κ = 0.76 for active TB findings. For findings consistent with TB, the sensitivity, specificity, and accuracy ranged from 77.2% to 91.1%, 87.4% to 98.6%, and 84.1% to 90.1%, respectively.

**Conclusion:**

B readers exhibited strong agreement and high accuracy in diagnosing TB on CXR, providing a robust dataset that could be used for external validation of AI models in TB diagnosis.

**Critical relevance statement:**

With substantial inter-rater agreement and strong diagnostic performance, B readers’ interpretations of CXRs provide a reliable dataset to support external validation of AI models for TB diagnosis.

**Key Points:**

- B readers can provide a reliable reference for validating AI models in TB diagnosis.
- Less variability and high accuracy were observed among B readers in CXR-based TB diagnosis.

**Graphical Abstract:**

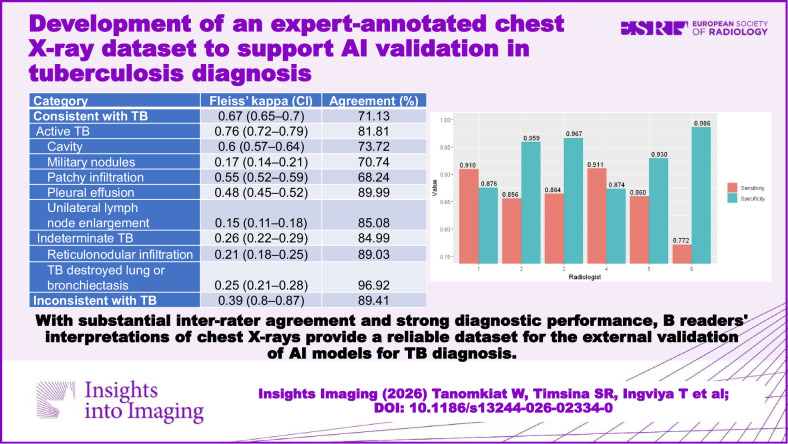

## Introduction

In 2022, an estimated 10.6 million people developed tuberculosis (TB), with approximately 1.3 million deaths attributed to this disease. Despite global efforts, TB-related deaths have decreased by only 19% from 2015 to 2022, far below the World Health Organization’s (WHO) target of a 75% reduction by 2025. This highlights the urgent need for intensified efforts and innovative strategies to combat TB effectively [1]. Eliminating TB is a key goal under the United Nations Sustainable Development Goals for Communicable Diseases, with a target of reducing TB deaths by 95% by 2035 through the End TB Strategy [1, 2]. A survey conducted in Thailand highlighted the high burden of TB, with a prevalence of 242 cases per 100,000 adults, emphasizing the need for the widespread utilization of chest X-ray (CXR) and other molecular technologies to enhance detection rates [3].

CXR is a WHO-recommended tool for systematic TB screening [4]. However, interpreting TB findings on CXR often involves inter-rater variability, with studies reporting only fair agreement among readers, particularly across different specialties or levels of experience [5–8]. Although CXR exhibits high sensitivity for TB diagnosis of TB, its specificity tends to be low [9, 10]. This is because CXR detects various lung abnormalities that are not always specific to TB, leading to variability in interpretation and an increased likelihood of false positives [11]. Despite these limitations, CXR remains a valuable tool for TB screening, especially with the advancement of artificial intelligence (AI)-based computer-aided detection (CAD) software.

In 2021, the WHO recommended the use of CAD software for TB screening and triage, renewing interest in CXR as a screening tool [4]. Many AI models have shown promising accuracy in TB screening [10, 12]. These AI models are rapidly entering markets like Thailand, where a shortage of radiologists and a high TB burden make them valuable for screening. However, studies have shown that many AI models perform worse on external datasets than on manufacturers’ internal data, with only 6% of the AI models in medical imaging being externally validated [13, 14]. Additionally, the lack of robust external datasets for validation limits their widespread use.

B readers are physicians certified by the National Institute for Occupational Safety and Health (NIOSH) for diagnosing pneumoconiosis and are considered skilled in interpreting CXRs. They demonstrate greater concordance with the final diagnosis of pneumoconiosis than other readers [15]. Given their expertise, we hypothesized that B readers would also demonstrate proficiency in identifying TB on CXR. Therefore, we aimed to assess the inter-rater agreement among six B readers in diagnosing TB on CXR and compare their performance with that of microbiologically confirmed TB. Additionally, the goal was to develop a reliable dataset to support external validation of AI models in TB detection.

## Methods

### Dataset

**Fig. 1:**
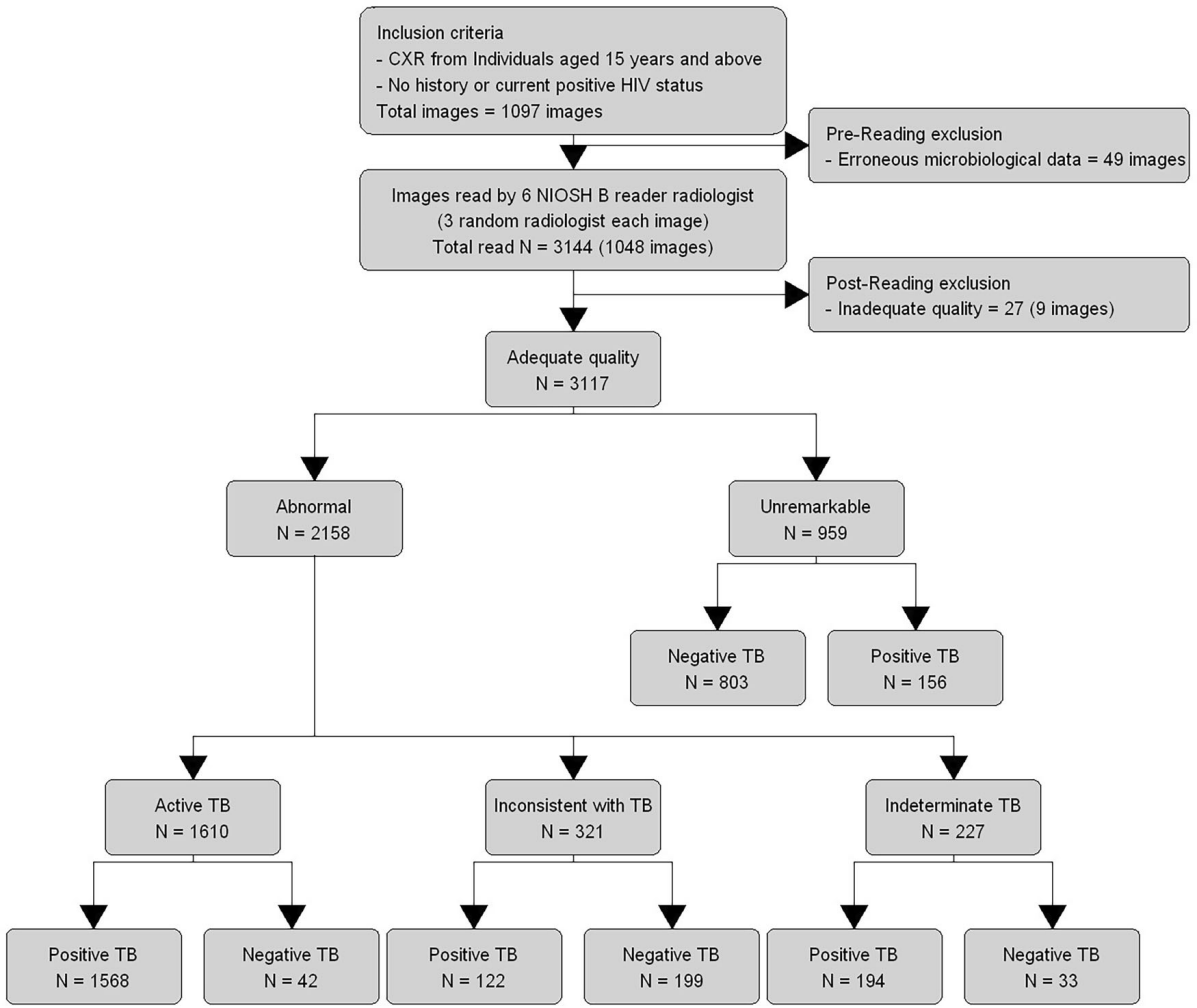
Flow chart of image readings included in the study (Positive TB = microbiologically positive for TB, Negative TB = microbiologically negative for TB). TB, tuberculosis

The dataset comprised 1097 CXRs that were carefully curated from five geographic locations in Thailand (Table 1). CXRs of individuals aged ≥ 15 years were included, excluding those with positive human immunodeficiency virus (HIV) serology. Data collection dates were unavailable because the digital imaging and communications in medicine (DICOM) metadata had been removed for de-identification. However, each institution was asked to provide CXRs taken between January 1, 2015, and December 31, 2020. Approximately 3144 readings of these CXRs were conducted by B readers in 2021. Owing to erroneous microbiological results, 49 images were excluded from the analysis. Of these, 3117 readings had adequate quality, whereas 27 readings (from nine images) were excluded because of inadequate image quality (Fig. 1). The study was approved by the Human Research Ethics Committees of the Faculty of Medicine, Prince of Songkla University (REC 61-424-9-1); Chiangrai Prachanukroh Hospital (EC CRH 050/65 Ex); and Mahasarakham University (330-340/2565). Informed consent was waived due to the retrospective nature of the data.

**Table 1:** Baseline characteristics

Characteristic	Center 1	Center 2	Center 3	Center 4	Center 5	Total
Total	300	298	200	149	150	1097
Sex						
Male	150 (50.0%)	172 (57.7%)	142 (71.0%)	88 (59.1%)	81 (54.0%)	633 (57.7%)
Female	150 (50.0%)	126 (42.3%)	58 (29.0%)	61 (40.9%)	69 (46.0%)	464 (42.3%)
Age (years)						
Mean (SD)	50.1 (17.4)	48.0 (15.9)	52.7 (15.3)	47.1 (17.0)	49.0 (17.9)	49.4 (16.7)
Median [IQR]	51.0 [37.0–63.5]	48.0 [36.2–60.0]	54.0 [43.0–66.0]	47.5 [34.0–61.0]	49.0 [35.0–62.8]	50.0 [37.0–62.0]
Range	[18–86]	[15–89]	[16–84]	[15–81]	[15–83]	[15–89]
Missing	9	20	13	5	8	55
Group						
TB	150	148	200	74	74	646
Non-TB	150	150	0	75	76	451

Center 1: Songklanagarind Hospital, Songkhla Province, Southern Thailand; Center 2: Chiangrai Prachanukroh Hospital, Chiang Rai Province, Northern Thailand; Center 3: Udon Thani Hospital, Udon Thani Province, Northeastern Thailand; Center 4: Suddhavej Hospital, Maha Sarakham Province, Northeastern Thailand; and Center 5: Division of Tuberculosis, Department of Disease Control, Ministry of Public Health, Central Thailand

*TB* tuberculosis, *SD* standard deviation, *IQR* interquartile range

**Fig. 2:**
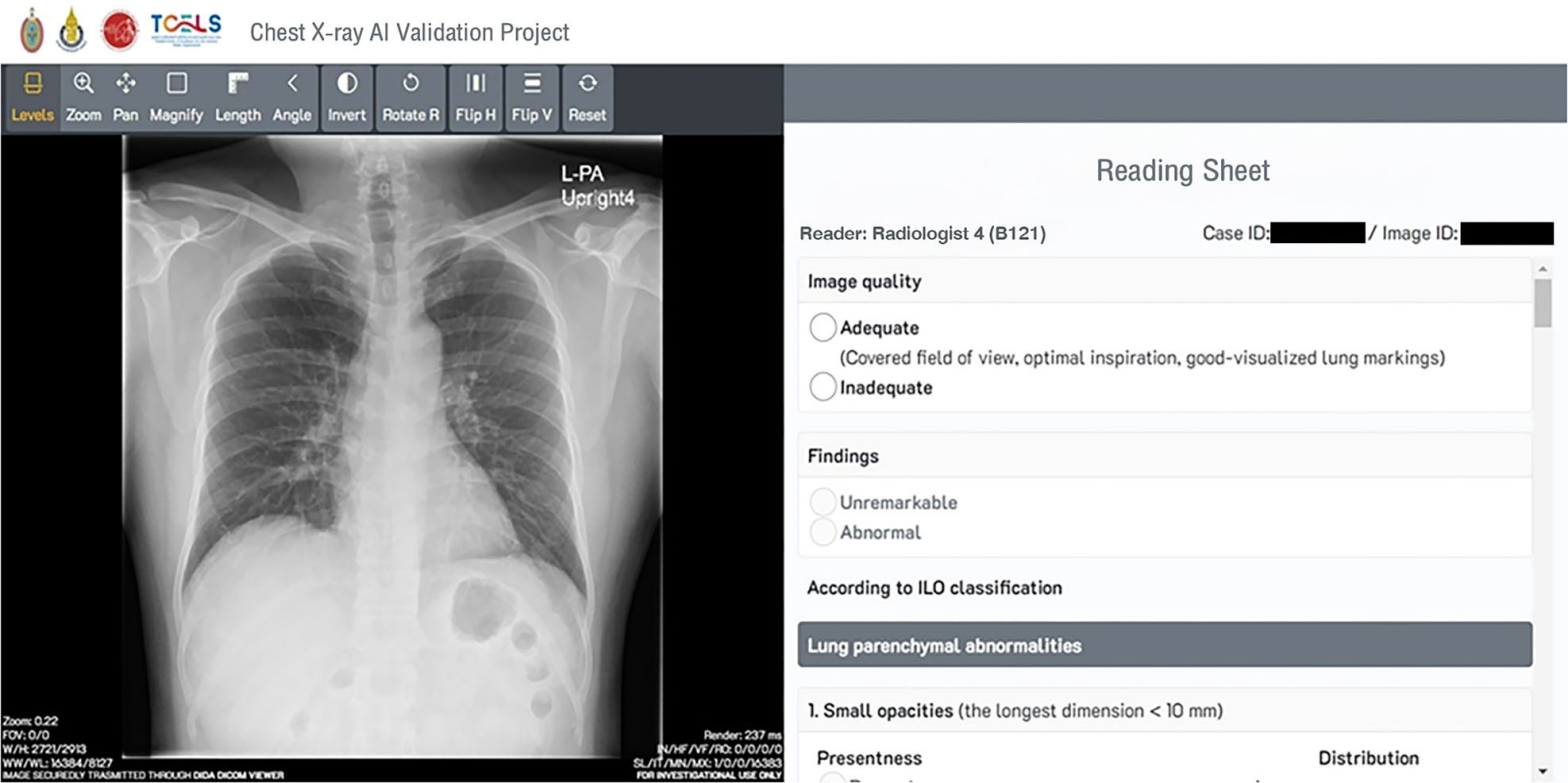
Web-based application integrating with the ePAD imaging platform and eCRF for data collection and management. eCRF, electronic case report form

CXRs were taken by FujiFilm®, Phillips®, Samsung®, or Siemen® digital imaging devices and exported from Synapse® picture archiving and communication system (PACS) as a DICOM file with 16-bit depth and resolution ranging from 2140 × 1760 to 4280 × 3520 pixels. All CXRs were uploaded to a web-based application equipped with an ePAD imaging platform and an electronic case report form (eCRF) (https://epad.stanford.edu). The ePAD platform, developed by the Rubin Lab at Stanford Medicine Radiology, functioned as a research PACS, whereas the integrated eCRF facilitated structured data collection and management (Fig. 2).

### B readers and data recording

B readers are physicians certified by the NIOSH who demonstrate proficiency in classifying radiographs of workers exposed to dust using the International Labor Organization (ILO) International Classification of Radiographs of Pneumoconiosis [16, 17]. They demonstrated greater concordance with the final pneumoconiosis diagnosis than the other readers [15]. B readers are certified through an exam assessing their ability to use the ILO classification system to evaluate and classify CXRs and must retest every 5 years to maintain certification [16].

**Fig. 3:**
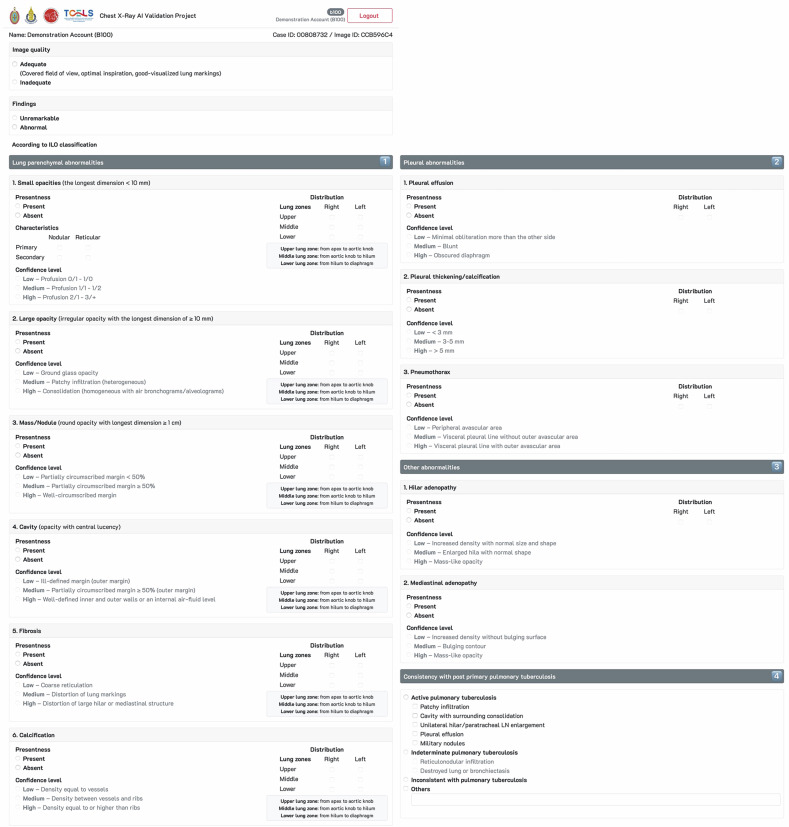
eCRF for data collection (modified from the reading sheet of the ILO). eCRF, electronic case report form; ILO, International Labour Organization

Six B readers were thoracic radiologists with over 15 years of experience: three with more than 30 years, two with 17 years, and one with 15 years of experience. Three of the B readers were from the same center, and the other three were from different centers. They were asked to interpret the CXRs and record their findings on reading sheets (Figs. 2 and 3) on the platform. After assessing the quality of the CXR, they classified each radiograph using a two-point scale to determine whether the images were unremarkable or showed abnormal findings. Abnormal findings were divided into three categories, modified from the ILO classification: (1) lung parenchymal abnormalities (small opacity, large opacity, mass/nodule, cavity, fibrosis, and calcification), (2) pleural abnormalities (pleural effusion, pleural thickening or calcification, and pneumothorax), and (3) other abnormalities (hilar adenopathy and mediastinal adenopathy) (Fig. 3). Finally, the CXRs were classified as either consistent (active pulmonary TB or indeterminate TB) or inconsistent with TB. Small opacity was defined as the longest dimension of < 1 cm and was subcategorized by shape as primary nodular, primary reticular, secondary nodular, or secondary reticular. The confidence level was graded on a three-point scale (profusion 1/0–1/2, score 1; 2/1–2/3, score 2; 3/2–3/+, score 3 according to the ILO classification). Cavity was defined as opacity with central lucency, and the confidence level was categorized into low (ill-defined outer margin), medium (partial circumscribed outer margin ≥ 50%), and high (well-defined inner and outer walls or an internal air-fluid level). The confidence level for fibrosis was categorized as low (coarse reticulation), medium (distortion of lung markings), or high (distortion of large hilar or mediastinal structures). The confidence level of calcification was categorized as low (density equal to the vessels), medium (density between the vessels and ribs), or high (density equal to or higher than the ribs). The confidence level of pleural effusion was categorized into low (minimal obliteration more than the other side), medium (blunt), and high (obscured diaphragm). The confidence level for pleural thickening or calcification was categorized as low (< 3 mm), medium (3–5 mm), or high (> 5 mm). The confidence level of pneumothorax was categorized as low (peripheral avascular area), medium (visceral pleural line without an outer avascular area), or high (visceral pleural line with an outer avascular area). The confidence level of the hilar adenopathy was categorized as low (increased density with normal size and shape), medium (enlarged hila with normal shape), or high (mass-like opacity). The confidence levels of mediastinal adenopathy were categorized as low (increased density without a bulging surface), medium (bulging contour), and high (mass-like opacity). Lung abnormalities were categorized into three zones: the upper zone (apex to aortic knob), middle zone (aortic knob to hilum), and lower zone (hilum to lung base).

Findings consistent with active TB included cavities, miliary nodules, patchy infiltration, pleural effusion, and unilateral lymph node enlargement [18]. Reticulonodular infiltration, destroyed lung, or bronchiectasis in the upper part of the lung without other findings were suggestive of TB with indeterminate activity.

### Microbiological reference

TB was confirmed in all cases using at least one diagnostic test: sputum smear (Ziehl–Neelsen), culture (Lowenstein–Jensen), or molecular testing for *Mycobacterium tuberculosis*.

### Statistical analysis

Descriptive statistics were used to summarize the data. Inter-rater agreement among the six B readers was assessed using the Fleiss’ kappa test and percentage agreement. Squared Cohen’s kappa was used to evaluate the agreement between pairs of radiologists with varying years of experience. Fleiss’ and Cohen’s κ values were interpreted as follows: κ < 0, poor agreement; κ = 0, no agreement; 0.01–0.20, slight agreement; 0.21–0.40, fair agreement; 0.41–0.60, moderate agreement; 0.61–0.80, substantial agreement; and 0.81–1.00, almost perfect agreement [19]. Per-image read counts (0–3), indicating how many readers identified each finding as present, were recorded. The final interpretation was defined by consensus (≥ 2 of 3 B readers).

For each B reader, contingency tables were constructed to compare the CXR findings with the microbiological results (Additional file 1: Table S8). The diagnostic performance of each B reader was evaluated using findings suggestive of post-primary TB (active and indeterminate TB) against microbiologically confirmed cases. Data and graphical figures were analyzed using R version 4.3.3.

## Results

### Baseline patient characteristics

Overall, 57.7% were male and 42.3% female with a mean age of 49.4 years (Table 1). Centers 1 and 2 contributed balanced cohorts with nearly equal numbers of TB and non-TB cases (150/150 and 148/150, respectively). Center 3 contributed only TB cases (200), while Centers 4 and 5 also included both groups in nearly equal proportions (74/75 and 74/76, respectively).

### All CXR findings

Of the 3117 readings, 69% (2158 readings) showed abnormal findings, whereas 31% (959 readings) were unremarkable (Table 2). The corresponding microbiological results confirmed that 87% (1884 readings) of the abnormal CXRs were from patients with TB, whereas 83% (803 readings) of the unremarkable CXRs were from non-TB patients. The number of interpretations varied among B readers, ranging from 452 to 587, while abnormal CXR findings were consistently identified across different interpretations, ranging from 66% to 73%.

**Table 2:** Findings from B readers’ interpretations across a spectrum of CXR findings and corresponding microbiological results

Characteristics	B readers’ interpretations							Microbiological results	
	1, *N* = 455	2, *N* = 452	3, *N* = 587	4, *N* = 585	5, *N* = 584	6, *N* = 454	All reads *N* = 3117	Negative TB, *N* = 1077	Positive TB, *N* = 2040
CXR findings, *n* (%)									
Abnormal	306 (67)	314 (69)	389 (66)	416 (71)	428 (73)	305 (67)	2158 (69)	274 (25)	1884 (92)
Unremarkable	149 (33)	138 (31)	198 (34)	169 (29)	156 (27)	149 (33)	959 (31)	803 (75)	156 (7.6)
Small opacity, *n* (%)	254 (56)	216 (48)	346 (59)	362 (62)	386 (66)	185 (41)	1749 (56)	136 (13)	1613 (79)
Large opacity, *n* (%)	238 (52)	264 (58)	308 (52)	335 (57)	265 (45)	241 (53)	1651 (53)	83 (7.7)	1568 (77)
Mass/nodule, *n* (%)	253 (56)	85 (19)	136 (23)	140 (24)	31 (5.3)	58 (13)	703 (23)	93 (8.6)	610 (30)
Cavity, *n* (%)	198 (44)	204 (45)	246 (42)	151 (26)	216 (37)	141 (31)	1156 (37)	18 (1.7)	1138 (56)
Fibrosis, *n* (%)	175 (38)	165 (37)	153 (26)	308 (53)	156 (27)	102 (22)	1059 (34)	62 (5.8)	997 (49)
Calcification, *n* (%)	39 (8.6)	72 (16)	14 (2.4)	243 (42)	30 (5.1)	19 (4.2)	417 (13)	40 (3.7)	377 (18)
Pleural effusion, *n* (%)	72 (16)	56 (12)	99 (17)	58 (9.9)	88 (15)	62 (14)	435 (14)	44 (4.1)	391 (19)
Pleural thickening, *n* (%)	166 (36)	137 (30)	143 (24)	114 (19)	129 (22)	55 (12)	744 (24)	47 (4.4)	697 (34)
Pneumothorax, *n* (%)	2 (0.4)	4 (0.9)	2 (0.3)	3 (0.5)	5 (0.9)	3 (0.7)	19 (0.6)	3 (0.3)	16 (0.8)
Hilar adenopathy, *n* (%)	100 (22)	43 (9.5)	52 (8.9)	143 (24)	20 (3.4)	67 (15)	425 (14)	46 (4.3)	379 (19)
Mediastinal adenopathy, *n* (%)	19 (4.2)	15 (3.3)	12 (2.0)	58 (9.9)	8 (1.4)	14 (3.1)	126 (4.0)	18 (1.7)	108 (5.3)
Consistent with TB	300 (66.4)	267 (59.1)	332 (56.6)	365 (62.9)	334 (57)	239 (52.6)	1837 (59.3)	75 (7)	1762 (86.5)
Active TB	280 (62)	244 (54)	305 (52)	313 (54)	259 (44)	209 (46)	1610 (52)	42 (3.9)	1568 (77)
Cavity, *n* (%)	195 (43)	190 (42)	229 (39)	110 (19)	177 (30)	118 (26)	1019 (33)	9 (0.8)	1010 (50)
Military nodules, *n* (%)	234 (51)	52 (12)	109 (19)	7 (1.2)	8 (1.4)	16 (3.5)	426 (14)	10 (0.9)	416 (20)
Patchy infiltration, *n* (%)	213 (47)	137 (30)	238 (41)	242 (41)	165 (28)	190 (42)	1185 (38)	18 (1.7)	1167 (57)
Pleural effusion, *n* (%)	67 (15)	32 (7.1)	32 (5.5)	25 (4.3)	27 (4.6)	33 (7.3)	216 (6.9)	8 (0.7)	208 (10)
Unilateral enlarged lymph node *n* (%)	95 (21)	21 (4.6)	17 (2.9)	12 (2.1)	10 (1.7)	39 (8.6)	194 (6.2)	13 (1.2)	181 (8.9)
Indeterminate TB	20 (4.4)	23 (5.1)	27 (4.6)	52 (8.9)	75 (13)	30 (6.6)	227 (7.3)	33 (3.1)	194 (9.5)
Reticulonodular infiltration, *n* (%)	0 (0)	12 (2.7)	26 (4.4)	37 (6.3)	61 (10)	16 (3.5)	152 (4.9)	21 (1.9)	131 (6.4)
Destroyed lung or bronchiectasis, *n* (%)	2 (0.4)	10 (2.2)	2 (0.3)	13 (2.2)	5 (0.9)	11 (2.4)	43 (1.4)	0 (0)	43 (2.1)
Inconsistent with TB Microbiological results, *n* (%)	6 (1.3)	47 (10)	57 (9.7)	51 (8.7)	94 (16)	66 (15)	321 (10)	199 (18)	122 (6.0)
Negative TB	145 (32)	147 (33)	211 (36)	214 (37)	213 (36)	147 (32)			
Positive TB	310 (68)	305 (67)	376 (64)	371 (63)	371 (64)	307 (68)			

*CXR* chest X-ray, *TB* tuberculosis

### Findings consistent with TB

Interpretations consistent with TB ranged from 52.6% to 66.4%. Within this range, 44%–62% of cases were classified as active TB, characterized by cavities (19%–50%), military nodules (1.2%–51%), patchy infiltration (28%–57%), pleural effusion (4.3% to 15%), and unilateral enlarged lymph nodes (1.7%–21%) (Table 2).

### Inter-rater agreement

Detection of all findings showed almost perfect agreement (Fleiss’ κ = 0.83, 95% confidence interval [CI]: 0.8–0.87; agreement = 89.41%) (Table 3). Interpretations consistent with TB and active TB had substantial agreement (Fleiss’ κ = 0.67, 95% CI: 0.65–0.7; agreement = 71.13% and Fleiss’ κ = 0.76, 95% CI: 0.72–0.79; agreement = 81.81%, respectively). Lowest agreement was for miliary nodules (Fleiss’ κ = 0.17, agreement = 70.74%).

**Table 3:** Fleiss’ kappa and percentage agreement for all 6 B readers

Category	Fleiss’ kappa (CI)	Agreement (%)
All findings	0.83 (0.8–0.87)	89.41
Small opacity	0.67 (0.63–0.7)	75.26
Large opacity	0.76 (0.72–0.79)	81.91
Mass/nodule	0.32 (0.28–0.35)	64.29
Cavity	0.67 (0.64–0.71)	77.19
Fibrosis	0.44 (0.4–0.47)	62.27
Calcification	0.16 (0.12–0.19)	70.74
Pleural effusion	0.71 (0.68–0.75)	89.61
Pneumothorax	0.68 (0.65–0.72)	99.42
Hilar adenopathy	0.33 (0.29–0.36)	76.32
Mediastinal adenopathy	0.2 (0.16–0.23)	90.66
Consistent with TB	0.67 (0.65–0.7)	71.13
Active TB	0.76 (0.72–0.79)	81.81
Cavity	0.6 (0.57–0.64)	73.72
Miliary nodules	0.17 (0.14–0.21)	70.74
Patchy infiltration	0.55 (0.52–0.59)	68.24
Pleural effusion	0.48 (0.45–0.52)	89.99
Unilateral lymph node enlargement	0.15 (0.11–0.18)	85.08
Indeterminate TB	0.26(0.22–0.29)	84.99
Reticulonodular infiltration	0.21 (0.18–0.25)	89.03
TB destroyed lung or bronchiectasis	0.25 (0.21–0.28)	96.92
Inconsistent with TB	0.39 (0.8–0.87)	89.41

*TB* tuberculosis

**Fig. 4:**
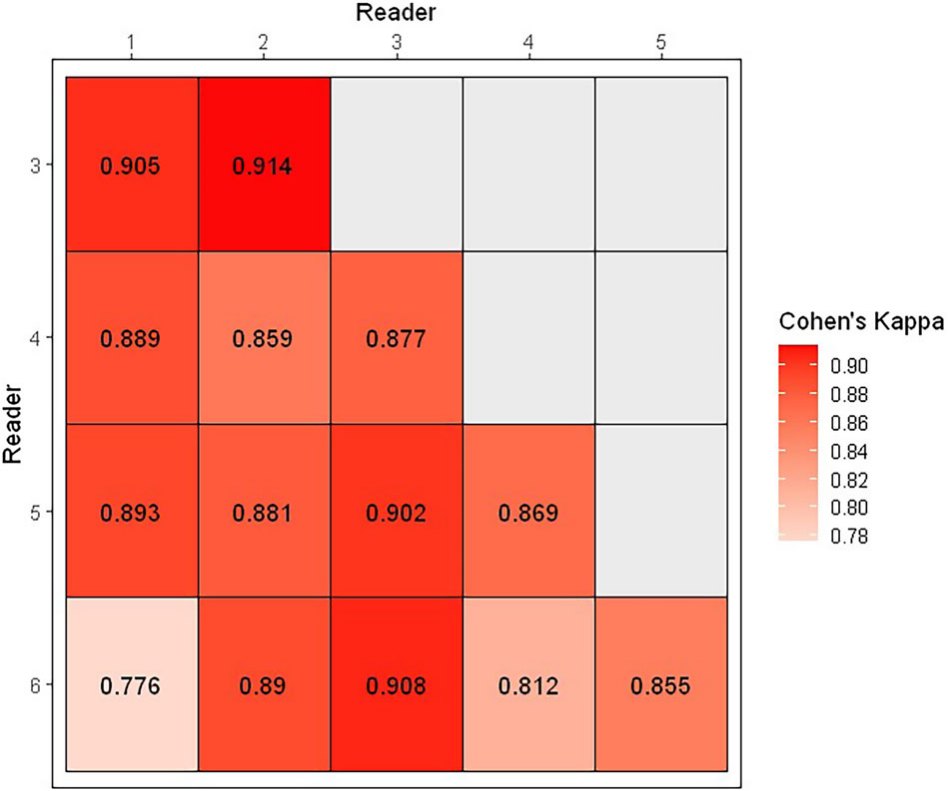
Heatmap showing Squared Cohen kappa for pairs of B readers with varying years of experience for CXR findings consistent with TB. CXR, chest X-ray; TB, tuberculosis

For CXR findings consistent with TB, the inter-rater agreement among pairs of B readers with varying years of experience showed the highest agreement between B reader 2 and B reader 3(Cohen’s κ = 0.91), whereas the lowest agreement was between B reader 1 and B reader 6 (Cohen’s κ = 0.78) (Fig. 4).

### Per-image read count analysis

All findings were stratified by microbiological TB status and per-image reader counts (0–3) (Table 4). For TB-consistent findings, > 94% of non-TB images (*n* = 359) received a 0-reader score. In contrast, TB-positive CXRs showed a substantial proportion of findings marked by ≥ 2 readers. For example, cavities were absent in nearly all non-TB cases (> 98% with 0 readers, 1.4% with 1 reader, and 0.6% with 2 readers), whereas among TB cases, they were identified by all three readers in 29% and by two readers in 22%. Among active TB findings, cavities had the highest consensus (≥ 2 readers: 51%; 346 cases), whereas unilateral lymph node enlargement had the lowest (4.6%; 31 cases). Complete results are available in Additional file 1 (Tables S1–S7) and Additional file 2.

**Table 4:** Per-image read counts distribution (0–3) for findings consistent with TB (active and indeterminate TB) on CXR, stratified by microbiological results

CXR findings	Non-TB *N* = 359	TB *N* = 680
Active TB		
Cavity, *n* (%)		
0	352 (98)	215 (32)
1	5 (1.4)	119 (18)
2	2 (0.6)	147 (22)
3	0 (0)	199 (29)
Military nodules, *n* (%)		
0	351 (98)	371 (55)
1	6 (1.7)	215 (32)
2	2 (0.6)	81 (12)
3	0 (0)	13 (1.9)
Patchy infiltration, *n* (%)		
0	343 (96)	149 (22)
1	14 (3.9)	112 (16)
2	2 (0.6)	202 (30)
3	0 (0)	217 (32)
Pleural effusion, *n* (%)		
0	352 (98)	558 (82)
1	6 (1.7)	61 (9.0)
2	1 (0.3)	36 (5.3)
3	0 (0)	25 (3.7)
Unilateral lymph node enlargement, *n* (%)		
0	350 (97)	532 (78)
1	5 (1.4)	117 (17)
2	4 (1.1)	29 (4.3)
3	0 (0)	2 (0.3)
Indeterminate TB		
Reticulonodular infiltration, *n* (%)		
0	339 (94)	581 (85)
1	19 (5.3)	72 (11)
2	1 (0.3)	22 (3.2)
3	0 (0)	5 (0.7)
Destroyed lung, *n* (%)		
0	359 (100)	646 (95)
1	0 (0)	27 (4.0)
2	0 (0)	5 (0.7)
3	0 (0)	2 (0.3)

Scores: 0 = no reader reported the finding, 1 = reported by 1 reader, 2 = reported by 2 readers, and 3 = reported by all 3 readers

Note: distribution of TB-consistent findings is shown; complete results for all other findings are available in additional file 1: Tables S1–S7

*CXR* chest X-ray, *TB* tuberculosis

### Diagnostic performances

**Fig. 5:**
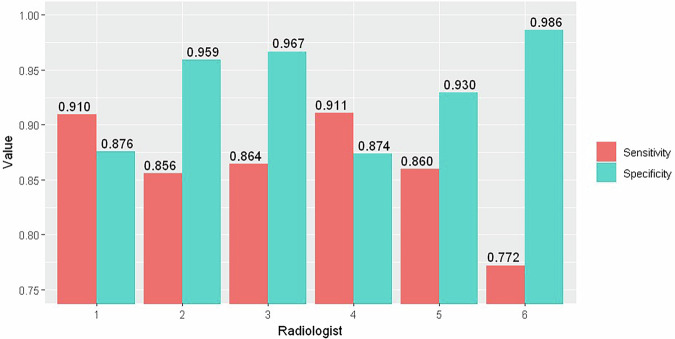
Diagnostic performance of six B readers in detecting TB on CXRs. Sensitivity and specificity are shown for each reader. CXR, chest X-ray

Diagnostic performance across the six B readers showed sensitivity, specificity, and accuracy ranging from 77.2%–91.1%, 87.4%–98.6%, and 84.1%–90.1%, respectively (Fig. 5 and Table 5).

**Table 5:** Diagnostic test metrics of B readers against microbiological results

	B reader 1	B reader 2	B reader 3	B reader 4	B reader 5	B reader 6
Total	455	452	587	585	584	454
TP	282	261	325	338	319	237
FP	18	6	7	27	15	2
TN	127	141	204	187	198	145
FN	28	44	51	33	52	70
Sensitivity	0.91	0.856	0.864	0.911	0.86	0.772
Specificity	0.876	0.959	0.967	0.874	0.93	0.986
Accuracy	0.899	0.889	0.901	0.897	0.885	0.841
AUC	0.893	0.907	0.916	0.892	0.895	0.879

Note: Metrics were calculated for findings consistent with TB (active + indeterminate TB)

*AUC* area under the curve, *TB* tuberculosis

## Discussion

This study is the first to assess the inter-rater variability and diagnostic performance among B readers for TB diagnosis using CXRs. The results showed almost perfect overall agreement (κ = 0.83) for all CXR findings, with substantial agreement on TB-specific findings (κ = 0.67). B readers demonstrated high sensitivity (77.2%–91.1%) and specificity (87.4%–98.6%) compared to the microbiological reference standard. Notably, their high specificity and consistent interpretation reduce overdiagnosis and improve CXR-based TB diagnosis.

Evaluating inter-rater variability is crucial because the diagnostic accuracy of CXR in identifying TB varies among readers [4–7]. In this study, a substantial agreement was noted in detecting findings consistent with TB with less variability compared with that in a previous study (κ = 0.67 vs 0.53) [5] (Table 3). The agreement in detecting active TB on CXR was considerable (κ = 0.76), surpassing the agreement levels reported in previous research (κ = 0.54–0.64) [5, 6]. Notably, reader experience may also influence inter-rater variation [6, 20]. Reportedly, with each additional 10 years of experience in a specialty, the diagnostic odds ratio for identifying TB increased 1.23-fold [6]. However, agreement on CXR findings consistent with TB among B readers remained substantial to almost perfect (κ = 0.78–0.91) (Fig. 4), suggesting that experience did not significantly impact the level of concordance.

The agreement for the detection of cavities and patchy infiltration, was comparable to that of previous studies [6, 8]. However, in the active TB category, the kappa value for pleural effusion dropped from 0.71 (considering all findings) to 0.48. This reduction may be due to readers making alternative diagnoses based on other features that are inconsistent with TB, leading to a lower agreement compared to that observed in earlier reports [6]. There was fair agreement regarding the detection of miliary nodules and lymph node enlargement. For miliary nodules, the agreement was lower than that noted in a previous study, which reported high agreement among radiologists in assessing miliary nodules in confirmed miliary TB cases [21]. In contrast, studies evaluating multiple CXR features of TB have shown low to fair agreement [7, 8, 22]. The lower agreement in our study may be due to the evaluation of a broader range of radiographic features of TB. Moreover, their small size and diffuse nature make detection challenging, with up to 50% of miliary TB cases diagnosed at autopsy [23]. Lymph node enlargement presents with subtle and often indistinct radiographic features, associated with anatomical complexities, and overlaps with other conditions. Previous studies have reported similar inter-rater variations and lower detection rates for mediastinal lymph nodes [22, 24].

Diagnosis of TB using CXR generally shows high sensitivity and low specificity [9–11]. However, studies from high-endemic areas like Kenya and Uganda have reported comparatively higher specificities of 67% and 97%, along with sensitivities of 91% and 93%, respectively [18, 25]. Similarly, in this study, higher specificity was observed, ranging from 87% to 98.6%, with slightly lower sensitivity, ranging from 77.2% to 91.1%, which may be attributed to a trade-off for high specificity. In comparison to our study, which specifically addressed inter-rater variation, the objectives of the aforementioned studies were different, and they did not report such variability. Diagnostic metrics may differ due to radiologists’ experience or the microbiological tests used. Two previous studies comparing AI models with human-read CXR reported varying diagnostic metrics among radiologists (sensitivity: 94.0% vs 98.3%; specificity: 45.6% vs 13.7%) [26, 27]. The first study used sputum culture as the microbiological reference, with CXRs read by International Organization for Migration (IOM)-certified radiologists with over 6 years of experience [26]. The second study used either sputum culture or polymerase chain reaction as a microbiological reference, with CXRs read by IOM radiologists with > 10 years of experience [27]. In contrast, in our study, readers maintained high diagnostic accuracy regardless of experience.

Amid the growing adoption of AI models for TB diagnosis, many AI models have demonstrated significant potential for TB diagnosis [10, 12, 26, 27]. However, studies have shown that AI models for radiological diagnosis often underperform on external datasets, a challenge that may arise from factors such as selection bias and overfitting [28, 29]. Although the exact reasons for the reduced performance of external data remain unclear [13], concerns have been raised about the reliability of using human-read CXRs as a reference standard, particularly when details about reader training, experience, and interrater variability have not been reported [30]. AI models often exhibit higher specificity when compared to human-read CXRs; however, its specificity tends to decrease when compared with that of microbiological tests. This could be due to the lower efficiency of human readers in identifying negative cases [31]. Unlike microbiological tests that yield binary results, CXRs reveal a spectrum of radiological abnormalities with varying clinical implications for disease stage, infectivity, treatment response, and prognosis. For instance, cavitary lesions are linked to higher bacterial loads and poorer outcomes [8]. Thus, detailed segmentation of these findings is essential for the meaningful validation of AI models. B readers’ interpretation provides a reliable reference for external validation of AI models. These robust datasets will be used to validate AI models for CXR-based TB diagnosis in Thailand.

The strengths of this study include its validation method for TB diagnosis: all cases were confirmed with microbiological testing, enhancing the validity of the TB diagnosis. In addition, to minimize subjective variation, each CXR was interpreted by three B readers, with ≥ 2 of 3 reads defining the consensus, which served as the final interpretation. By involving multiple centers across Thailand, we incorporated diverse cases, reducing potential selection bias related to patterns from any single site.

This study has some limitations. We excluded HIV-positive and pediatric CXRs and drew data from high-incidence regions of Thailand; therefore, our findings may not apply to these patient groups or to low-incidence settings. No comparisons between B and non-B readers were conducted, limiting broader insights into inter-rater variability; future studies should assess agreement between these groups.

In conclusion, our findings demonstrated that B readers exhibit high accuracy and consistency in diagnosing TB on CXR. Despite the emerging use of AI models in TB diagnosis, their real-world performance and clinical utility vary by country and region. This dataset offers a reliable reference for external validation of AI models in TB diagnosis.

## Supplementary information

ELECTRONIC SUPPLEMENTARY MATERIAL

Supplementary Data

## Data Availability

The data used in the study are available from the corresponding author upon request.

## Abbreviations

AI: Artificial intelligence
CAD: Computer-aided detection
CI: Confidence interval
CXR: Chest X-ray
DICOM: Digital imaging and communications in medicine
eCRF: Electronic case report form
HIV: Human immunodeficiency virus
ILO: International Labor Organization
IOM: International organization for migration
NIOSH: National Institute for Occupational Safety and Health
PACS: Picture archiving and communication system
TB: Tuberculosis
WHO: World Health Organization
