# Supplementary material for: Development of an expert-annotated chest X-ray dataset to support AI validation in tuberculosis diagnosis
Source: Insights Imaging. 2026 Jul 10;17:183. doi: 10.1186/s13244-026-02334-0 (PMC13354736; doi:10.1186/s13244-026-02334-0)
Supplement: Supplementary file 1 — ELECTRONIC SUPPLEMENTARY MATERIAL [file 13244_2026_2334_MOESM1_ESM.pdf]

Development of an expert-annotated chest X-ray dataset to support AI validation in tuberculosis diagnosis

ELECTRONIC SUPPLEMENTARY MATERIAL

Table S1. Per image read counts distribution (0–3) for small opacity detected on CXR, stratified by microbiological results

Number of "Positive" read among 3 B readers	**Non-TB** N = 359	**TB** N = 680
Small opacity, n (%)		
0	259 (72)	71 (10)
1	73 (20)	48 (7.1)
2	18 (5.0)	118 (17)
3	9 (2.5)	443 (65)
Small opacity primary nodular, n (%)		
0	327 (91)	135 (20)
1	24 (6.7)	114 (17)
2	4 (1.1)	171 (25)
3	4 (1.1)	260 (38)
Small opacity secondary nodular, n (%)		
0	314 (87)	127 (19)
1	38 (11)	233 (34)
2	4 (1.1)	244 (36)
3	3 (0.8)	76 (11)
Small opacity primary reticular, n (%)		
0	283 (79)	410 (60)
1	62 (17)	193 (28)
2	12 (3.3)	61 (9.0)
3	2 (0.6)	16 (2.4)
Small opacity secondary reticular, n (%)		
0	290 (81)	284 (42)
1	61 (17)	256 (38)
2	7 (1.9)	120 (18)
3	1 (0.3)	20 (2.9)
Small opacity lower left, n (%)		
0	306 (85)	342 (50)
1	45 (13)	110 (16)
2	3 (0.8)	101 (15)
3	5 (1.4)	127 (19)
Small opacity lower right, n (%)		
0	284 (79)	358 (53)
1	57 (16)	101 (15)
2	10 (2.8)	92 (14)
3	8 (2.2)	129 (19)
Small opacity middle left, n (%)		
0	333 (93)	217 (32)
1	18 (5.0)	66 (9.7)
2	4 (1.1)	127 (19)
3	4 (1.1)	270 (40)
Small opacity middle right, n (%)		

0	314 (87)	168 (25)
1	31 (8.6)	93 (14)
2	8 (2.2)	122 (18)
3	6 (1.7)	297 (44)
Small opacity upper left, n (%)		
0	343 (96)	285 (42)
1	13 (3.6)	101 (15)
2	1 (0.3)	83 (12)
3	2 (0.6)	211 (31)
Small opacity upper right, n (%)		
0	340 (95)	236 (35)
1	13 (3.6)	88 (13)
2	3 (0.8)	114 (17)
3	3 (0.8)	242 (36)

Scores: 0 = no reader reported the finding, 1 = reported by 1 reader, 2 = reported by 2 readers, 3 = reported by all 3 readers

Right = right lung; Left = left lung

Lung zones: upper (apex to aortic knob), middle (aortic knob to inferior aspect of hilum) and lower (hilum to lung base)

Abbreviations: CXR, chest X-ray; TB, tuberculosis

Table S2. Per image read counts distribution (0–3) for large opacity detected on CXR, stratified by microbiological results

Number of "Positive" read among 3 B readers	**Non-TB** N = 359	**TB** N = 680
Large opacity, n (%)		
0	314 (87)	88 (13)
1	20 (5.6)	52 (7.6)
2	12 (3.3)	104 (15)
3	13 (3.6)	436 (64)
Large opacity lower left, n (%)		
0	343 (96)	468 (69)
1	9 (2.5)	91 (13)
2	3 (0.8)	67 (9.9)
3	4 (1.1)	54 (7.9)
Large opacity lower right, n (%)		
0	336 (94)	482 (71)
1	13 (3.6)	81 (12)
2	7 (1.9)	56 (8.2)
3	3 (0.8)	61 (9.0)
Large opacity middle left, n (%)		
0	348 (97)	333 (49)
1	7 (1.9)	95 (14)
2	2 (0.6)	83 (12)
3	2 (0.6)	169 (25)
Large opacity middle right, n (%)		
0	334 (93)	302 (44)
1	12 (3.3)	114 (17)
2	4 (1.1)	99 (15)
3	9 (2.5)	165 (24)
Large opacity upper left, n (%)		
0	355 (99)	421 (62)
1	3 (0.8)	83 (12)
2	0 (0)	62 (9.1)
3	1 (0.3)	114 (17)
Large opacity upper right, n (%)		

0	351 (98)	347 (51)
1	4 (1.1)	90 (13)
2	2 (0.6)	111 (16)
3	2 (0.6)	132 (19)

Scores: 0 = no reader reported the finding, 1 = reported by 1 reader, 2 = reported by 2 readers, 3 = reported by all 3 readers
Right = right lung; Left = left lung
Lung zones: upper (apex to aortic knob), middle (aortic knob to inferior aspect of hilum) and lower (hilum to lung base)
Abbreviations: CXR, chest X-ray; TB, tuberculosis

Table S3. Per image read counts distribution (0–3) for mass/nodule detected on CXR, stratified by microbiological results

Number of "Positive" read among 3 B readers	**Non-TB** N = 359	**TB** N = 680
Mass/ nodule, n (%)		
0	318 (89)	282 (41)
1	9 (2.5)	234 (34)
2	12 (3.3)	116 (17)
3	20 (5.6)	48 (7.1)
Mass/nodule lower left, n (%)		
0	353 (98)	565 (83)
1	4 (1.1)	100 (15)
2	2 (0.6)	10 (1.5)
3	0 (0)	5 (0.7)
Mass/nodule lower right, n (%)		
0	347 (97)	554 (81)
1	9 (2.5)	108 (16)
2	3 (0.8)	10 (1.5)
3	0 (0)	8 (1.2)
Mass/nodule middle left, n (%)		
0	347 (97)	431 (63)
1	10 (2.8)	201 (30)
2	1 (0.3)	44 (6.5)
3	1 (0.3)	4 (0.6)
Mass/nodule middle right, n (%)		
0	333 (93)	423 (62)
1	6 (1.7)	194 (29)
2	12 (3.3)	50 (7.4)
3	8 (2.2)	13 (1.9)
Mass/nodule upper left, n (%)		
0	352 (98)	512 (75)
1	3 (0.8)	138 (20)
2	1 (0.3)	26 (3.8)
3	3 (0.8)	4 (0.6)
Mass/nodule upper right, n (%)		
0	349 (97)	480 (71)
1	6 (1.7)	177 (26)
2	2 (0.6)	15 (2.2)
3	2 (0.6)	8 (1.2)

Scores: 0 = no reader reported the finding, 1 = reported by 1 reader, 2 = reported by 2 readers, 3 = reported by all 3 readers
Right = right lung; Left = left lung
Lung zones: upper (apex to aortic knob), middle (aortic knob to inferior aspect of hilum) and lower (hilum to lung base)
Abbreviations: CXR, chest X-ray; TB, tuberculosis

Table S4. Per image read counts distribution (0–3) for cavity detected on CXR, stratified by microbiological results

Number of "Positive" read among 3 readers	**Non-TB** N = 359	**TB** N = 680
Cavity, n (%)		
0	347 (97)	189 (28)
1	8 (2.2)	108 (16)
2	2 (0.6)	119 (18)
3	2 (0.6)	264 (39)
Cavity lower left, n (%)		
0	357 (99)	638 (94)
1	1 (0.3)	28 (4.1)
2	1 (0.3)	12 (1.8)
3	0 (0)	2 (0.3)
Cavity lower right, n (%)		
0	357 (99)	637 (94)
1	0 (0)	28 (4.1)
2	0 (0)	6 (0.9)
3	2 (0.6)	9 (1.3)
Cavity middle left, n (%)		
0	357 (99)	458 (67)
1	2 (0.6)	98 (14)
2	0 (0)	72 (11)
3	0 (0)	52 (7.6)
Cavity middle right, n (%)		
0	353 (98)	439 (65)
1	5 (1.4)	109 (16)
2	1 (0.3)	53 (7.8)
3	0 (0)	79 (12)
Cavity upper left, n (%)		
0	359 (100)	503 (74)
1	0 (0)	75 (11)
2	0 (0)	49 (7.2)
3	0 (0)	53 (7.8)
Cavity upper right, n (%)		
0	357 (99)	440 (65)
1	2 (0.6)	107 (16)
2	0 (0)	57 (8.4)
3	0 (0)	76 (11)

Scores: 0 = no reader reported the finding, 1 = reported by 1 reader, 2 = reported by 2 readers, 3 = reported by all 3 readers
Right = right lung; Left = left lung
Lung zones: upper (apex to aortic knob), middle (aortic knob to inferior aspect of hilum) and lower (hilum to lung base)
Abbreviations: CXR, chest X-ray; TB, tuberculosis

Table S5. Per image read counts distribution (0–3) for fibrosis detected on CXR, stratified by microbiological results

Number of "Positive" read among 3 readers	**Non-TB**<br>N = 359	**TB**<br>N = 680
Fibrosis, n (%)		
0	314 (87)	172 (25)
1	29 (8.1)	179 (26)
2	15 (4.2)	169 (25)
3	1 (0.3)	160 (24)
Fibrosis lower left, n (%)		
0	347 (97)	607 (89)
1	9 (2.5)	52 (7.6)
2	3 (0.8)	15 (2.2)
3	0 (0)	6 (0.9)
Fibrosis lower right, n (%)		
0	345 (96)	612 (90)
1	8 (2.2)	48 (7.1)
2	6 (1.7)	12 (1.8)
3	0 (0)	8 (1.2)
Fibrosis middle left, n (%)		
0	353 (98)	434 (64)
1	4 (1.1)	121 (18)
2	2 (0.6)	86 (13)
3	0 (0)	39 (5.7)
Fibrosis middle right, n (%)		
0	349 (97)	416 (61)
1	9 (2.5)	144 (21)
2	1 (0.3)	81 (12)
3	0 (0)	39 (5.7)
Fibrosis upper left, n (%)		
0	354 (99)	443 (65)
1	5 (1.4)	118 (17)
2	0 (0)	73 (11)
3	0 (0)	46 (6.8)
Fibrosis upper right, n (%)		
0	346 (96)	365 (54)
1	8 (2.2)	138 (20)
2	4 (1.1)	129 (19)
3	1 (0.3)	48 (7.1)

Scores: 0 = no reader reported the finding, 1 = reported by 1 reader, 2 = reported by 2 readers, 3 = reported by all 3 readers
Right = right lung; Left = left lung
Lung zones: upper (apex to aortic knob), middle (aortic knob to inferior aspect of hilum) and lower (hilum to lung base)
Abbreviations: CXR, chest X-ray; TB, tuberculosis

Table S6. Per image read counts distribution (0–3) for pleural-based findings detected on CXR, stratified by microbiological results

Number of "Positive" read among 3 readers	**Non-TB**<br>N = 359	**TB**<br>N = 680
Pleural effusion, n (%)		
0	338 (94)	500 (74)
1	9 (2.5)	51 (7.5)
2	1 (0.3)	47 (6.9)
3	11 (3.1)	82 (12)
Pleural effusion left, n (%)		
0	349 (97)	573 (84)
1	4 (1.1)	31 (4.6)
2	0 (0)	32 (4.7)
3	6 (1.7)	44 (6.5)
Pleural effusion right, n (%)		
0	344 (96)	583 (86)
1	7 (1.9)	28 (4.1)
2	2 (0.6)	26 (3.8)
3	6 (1.7)	43 (6.3)
Pleural thickening, n (%)		
0	327 (91)	328 (48)
1	20 (5.6)	127 (19)
2	9 (2.5)	105 (15)
3	3 (0.8)	120 (18)
Pleural thickening left, n (%)		
0	340 (95)	439 (65)
1	16 (4.5)	124 (18)
2	1 (0.3)	62 (9.1)
3	2 (0.6)	55 (8.1)
Pleural thickening right, n (%)		
0	333 (93)	424 (62)
1	17 (4.7)	111 (16)
2	8 (2.2)	67 (9.9)
3	1 (0.3)	78 (11)
Pneumothorax, n (%)		
0	358 (100)	671 (99)
1	0 (0)	5 (0.7)
2	0 (0)	1 (0.1)
3	1 (0.3)	3 (0.4)
Pneumothorax left, n (%)		
0	359 (100)	676 (99)
1	0 (0)	1 (0.1)
2	0 (0)	1 (0.1)
3	0 (0)	2 (0.3)
Pneumothorax right, n (%)		
0	358 (100)	675 (99)
1	0 (0)	4 (0.6)
2	0 (0)	0 (0)
3	1 (0.3)	1 (0.1)

Scores: 0 = no reader reported the finding, 1 = reported by 1 reader, 2 = reported by 2 readers, 3 = reported by all 3 readers
Right = right lung; Left = left lung
Lung zones: upper (apex to aortic knob), middle (aortic knob to inferior aspect of hilum) and lower (hilum to lung base)

Abbreviations: CXR, chest X-ray; TB, tuberculosis

Table S7. Per image read counts distribution (0–3) for adenopathy detected on CXR, stratified by microbiological results

Number of "Positive" read among 3 readers	**Non-TB** N = 359	**TB** N = 680
Hilar adenopathy, n (%)		
0	331 (92)	425 (63)
1	16 (4.5)	162 (24)
2	6 (1.7)	62 (9.1)
3	6 (1.7)	31 (4.6)
Hilar adenopathy left, n (%)		
0	348 (97)	529 (78)
1	9 (2.5)	103 (15)
2	2 (0.6)	31 (4.6)
3	0 (0)	17 (2.5)
Hilar adenopathy right, n (%)		
0	337 (94)	540 (79)
1	12 (3.3)	92 (14)
2	4 (1.1)	31 (4.6)
3	6 (1.7)	17 (2.5)
Mediastinal adenopathy, n (%)		
0	348 (97)	590 (87)
1	6 (1.7)	74 (11)
2	3 (0.8)	14 (2.1)
3	2 (0.6)	2 (0.3)
Mediastinal adenopathy left, n (%)		
0	359 (100)	680 (100)
1	0 (0)	0 (0)
2	0 (0)	0 (0)
3	0 (0)	0 (0)
Mediastinal adenopathy right, n (%)		
0	359 (100)	680 (100)
1	0 (0)	0 (0)
2	0 (0)	0 (0)
3	0 (0)	0 (0)

Scores: 0 = no reader reported the finding, 1 = reported by 1 reader, 2 = reported by 2 readers, 3 = reported by all 3 readers
Right = right lung; Left = left lung
Lung zones: upper (apex to aortic knob), middle (aortic knob to inferior aspect of hilum) and lower (hilum to lung base)
Abbreviations: CXR, chest X-ray; TB, tuberculosis

Table S8. Contingency table showing CXR findings against microbiological results

	Microbiological results											
	B reader 1		B reader 2		B reader 3		B reader 4		B reader 5		B reader 6	
**CXR findings**	TB	Non-TB	TB	Non-TB	TB	Non-TB	TB	Non-TB	TB	Non-TB	TB	Non-TB
Consistent with TB	282	18	261	6	325	7	338	27	319	15	237	2
Active TB	269	11	240	4	299	6	301	12	252	7	207	2
Indeterminate TB	13	7	21	2	26	1	37	15	67	8	30	0
Inconsistent with TB	3	3	15	32	22	35	16	35	26	68	40	26
Unremarkable	25	124	29	109	29	169	17	152	26	130	30	119

Abbreviations: CXR, chest X-ray; TB, tuberculosis
